# K-RAS Is…Complicated

**DOI:** 10.3390/cancers15225480

**Published:** 2023-11-20

**Authors:** Geoffrey J. Clark

**Affiliations:** Department of Pharmacology & Toxicology, University of Louisville, Rm 417, CTRB, 505 S. Hancock St., Louisville, KY 40202, USA; gjclar01@louisville.edu

There is little argument that the K-RAS onco-protein is the most important single oncoprotein in human cancer. The frequency with which it is aberrantly activated by point mutations in its gene is the highest of all. These mutations almost always occur at codon 12, where virtually any amino acid substitution from the native Glycine leads to the potential for transformation [1,2].

Traditionally, K-RAS has been envisaged as a small, simple, on (GTP-bound)/off (GDP-bound) GTPase switch at the plasma membrane. Oncogenic mutations have the effect of stabilizing the active GTP-bound form or accelerating the GTP/GDP cycle, which have a similar net effect. In the active GTP-bound form, K-RAS regulates three mitogenic pathways: RAF/MAPK, PI3-Kinase and RALGEFs [3]. A few other proteins have been described as direct effectors, but in general have not been given a great deal of attention. Indeed, current mainstream opinion generally favors the concept that overstimulating the RAF/MAPK pathway is probably the most important function of K-RAS in cancer [4].

Recent proteomic studies have shown that, in fact, mutant K-RAS has the potential to directly bind and modulate over 30 proteins that impact diverse cellular processes and can be detected in stable complexes with over 1000 different proteins [5,6]. Different RAS mutants may exhibit differential protein association patterns. This confirms empirical results that different amino acid residues at the mutant hotspot codon 12 can lend the mutants significantly different biological effects [7]. Moreover, considerable evidence has been presented that suggests that K-RAS may be capable of forming nanoclusters essential for proper effector activation [8]. So, the function of K-RAS in tumors has the potential to be extremely complicated.

Until recently, elucidating the function of K-RAS was a fascinating and often frustrating academic exercise, but little more, because it was not directly actionable in the clinic. All attempts at directly targeting K-RAS with drugs had failed. This has now changed. By leveraging the ability of Cysteine residues to form covalent bonds, an effective inhibitor of the specific mutant isoform activated by a Cysteine mutation at codon 12 has been developed [9]. The binding of the drug traps this fast-cycling RAS mutant in the inactive, GDP-bound form. The approval of this first anti-RAS drug, designated Sotorasib, in 2021 [10] has led to a veritable stampede of RAS drug development. Now, over 20 companies and several academic laboratories have pre-clinical agents that cover all common forms of activated K-RAS. Therefore, understanding how K-RAS works in tumors and how the different anti-RAS drugs affect RAS function may be critical in optimizing their use in patients.

The recent manuscript from Nolan et al. [11] uses a proteomics approach to make several important contributions to the understanding of how K-RAS may function and how it responds to treatment with Sotorasib. The work confirms that different RAS mutants exhibit different protein-binding profiles, in addition the fact that the host cell type plays a major role in determining the proteins that RAS interacts with. They also show that non-mutated wild-type K-RAS has a large set of proteins with which it can complex that are quite different to those found in complexes with mutant K-RAS. This suggests that wild-type K-RAS may have its own “effector set” that only binds to the GDP-bound inactive form. This concept was raised years ago when it was shown that the transcription factor Aiolos can bind to wild-type but not mutant K-RAS and that wild-type K-RAS inhibits its function, perhaps by trapping it in the plasma membrane [12]. These observations may help provide a mechanistic explanation for the apparent tumor-suppressing effects of the wild-type allele. This area of RAS biology is underexplored.

The group also examines the effects of Sotorasib on the protein-binding profile of its susceptible target mutant K-RAS 12C, as well as wild-type K-Ras and two other activating mutants, which should be immune to the drug. It appears that the drug causes significant shifts in the protein interaction fingerprint of all the K-Ras proteins, even though it should not interact with the non-12C isoforms in a significant manner. These include increasing the interaction of wild-type K-RAS with src family kinases. The mechanisms and biological significance of these observations remain unclear. However, Sotorasib has already been shown to interact with over 130 off-target proteins, some of which, such as KEAP1, may be responsible for significant off-target biological effects [13].

Thus, the cellular response to the K-RAS 12C inhibitor Sotorasib is much more complex than originally conceived and this may have considerable ramifications for patient selection and the development of resistance. One possible source of error in Nolan et al.’s work is the relatively high levels of drug used in the experiments, but the potential for unintended effects on K-RAS is clear. It will be interesting to apply similar studies to primary patient samples from the clinic to determine how physiological the findings are and whether they can be leveraged to enhance therapeutic effectiveness.
